# Narratives of family members on the suicide of older adults in an Amazonian metropolis

**DOI:** 10.11606/S1518-8787.2017051007059

**Published:** 2017-12-04

**Authors:** André Luis Sales da Costa, Maximiliano Loiola Ponte de Souza

**Affiliations:** I Universidade Federal do Amazonas. Programa de Pós-Graduação em Saúde, Sociedade e Endemias na Amazônia. Manaus, AM, Brasil; II Fundação Oswaldo Cruz. Instituto Leônidas e Maria Deane. Manaus, AM, Brasil

**Keywords:** Aged, Suicide, psychology, Risk Factors, Personal Narratives, Hermeneutics, Idoso, Suicídio, psicologia, Fatores de Risco, Narrativas Pessoais, Hermenêutica

## Abstract

**OBJECTIVE:**

To analyze the narratives of family members on the suicide of older adults in Manaus, State of Amazonas, Brazil.

**METHODS:**

This is a qualitative study of the narratives of eight older adults, who committed suicide in the period of 2001-2012. In the analytic-interpretative process, we have tried to perform the hermeneutic double exercise: to interpret the interpretation of narrators. We have used as theoretical references authors who have investigated suicide from the perspective of gender and its correlations with the sociofamiliar context and with mental disorders.

**RESULTS:**

The family members would conceive the suicide of the older adults as related to losses, which would occur in a strained sociofamiliar scenario, leading to the appearance of psychopathological situations that, if not properly followed, would result in death. There would also be something inexorable in this sequence of events. The older adults, by the very time of their life, would tend to accumulate losses of different aspects in their trajectory. Their rigor and other relational limitations would simultaneously stress family relationships, favoring conflicts, and hinder adherence to treatment. This model of understanding, which has a wide support in the hegemonic medical-psychological discourse, in a sense minimizes possible self- or heteroaccusations directed at family members.

**CONCLUSIONS:**

Special attention should be given to identify the older adults who present losses, family conflicts, and signs of psychopathology and who do not follow-up psychosocial care services. Strategies to help older adults handle family conflicts and losses, empowering them, should be developed and made available by intersectoral actions. The adequate treatment of psychopathological conditions should be implanted in a context in which active search mechanisms also existed for older adults who abandoned follow-up. The implementation of these actions is a challenge to be faced in Manaus, State of Amazonas, Brazil, where there is a low availability of psychosocial care services, which are not articulated with specialized care services in tertiary medical conditions, and there is still low coverage by the basic care.

## RESUMO

**OBJETIVO:**

Analisar as narrativas de familiares sobre o suicídio de idosos em Manaus, Amazonas, Brasil.

**MÉTODOS:**

Estudo qualitativo de narrativas de oito idosos que morreram por suicídio no período 2001-2012. No processo analítico-interpretativo, buscou-se realizar o exercício da dupla hermenêutica: interpretar a interpretação dos narradores. Teve-se como referenciais teóricos autores que investigaram o suicídio a partir da perspectiva de gênero, de suas correlações com o contexto sociofamiliar e com transtornos mentais.

**RESULTADOS:**

Os familiares conceberiam o suicídio dos idosos como relacionado a perdas, que se dariam em um cenário sociofamiliar tensionado, levando ao surgimento de quadros psicopatológicos que, se não adequadamente acompanhados, resultariam na morte. Haveria também algo de inexorável nessa sequência de eventos. O idoso, pelo próprio tempo de sua vida, tenderia a acumular em sua trajetória perdas de diferentes matizes. Sua rigidez e outras limitações relacionais tensionariam simultaneamente as relações familiares, favorecendo conflitos, e dificultariam a adesão ao tratamento. Esse modelo de compreensão, que tem amplo ancoramento no discurso médico-psicológico hegemónico, de certo modo, se presta a minimizar eventuais auto- ou heteroacusações direcionadas aos familiares.

**CONCLUSÕES:**

Especial atenção deveria ser direcionada para se identificar idosos que apresentem simultaneamente perdas, conflitos familiares, indícios de psicopatologia e não seguimento em serviços de atenção psicossocial. Estratégias para auxiliar os idosos a lidar com as perdas e conflitos familiares, empoderando-os, deveriam ser desenvolvidas e disponibilizadas por meio de ações intersetoriais. O adequado tratamento de condições psicopatológicas deveria ser implantado, em um contexto no qual também houvesse mecanismos de busca ativa de idosos que abandonaram o acompanhamento. Implantar essas ações é um desafio a ser enfrentado em Manaus, onde há baixa disponibilidade de serviços de atenção psicossocial, que não se articulam com os serviços de atenção especializada em condições médicas de abordagem terciária, havendo ainda baixa cobertura pela atenção básica.

## INTRODUCTION

In 2020, more than 1.5 million persons in the world will commit suicide^2^. The magnitude of this value is possibly related to the process of population aging, since the group of older adults is the one that presents the greatest risk for suicide in the world^2^. In the most populous municipality of the northern region of Brazil, Manaus, capital of the state of Amazonas, older adults accounted for 4.7% of the population in 2000, and 6% in 2010^a^. In this period, in Manaus, the mortality rate for suicide in older adults was 4.5/100,000, a value 50% higher than that found in the state^b^.

Since the 1950s, studies have investigated the circumstances of suicide from standardized interviews with key informants (psychological autopsies). Over time, these instruments have become increasingly focused on the identification of psychiatric diagnoses and less interested in the social context of suicide^18^. In Brazil, an instrument called “Semistructured interview guide for psychological and psychosocial autopsies” (*“Roteiro de entrevista semiestruturada para autópsias psicológicas e psicossociais”)* has been developed for research among older adults^3^. Such instrument differs from classic psychological autopsies insofar as it rescues the importance of exploring social aspects and opens space for the subjectivity of the interviewees.

Owens and Lambert^18^ have observed that when persons are asked about the death of someone close to them, even if closed instruments are used, they end up interspersing their answers with narrative fragments about the circumstances of the death. Such materials are not usually explored, or there is no epistemic zeal to consider that they are strategies constructed to give sense, from the present, to past events^9^. It is a rich material to access an important and unexplored dimension of suicide.

Considering the importance of producing qualitative information on the suicide of older adults in poorly explored contexts, the purpose of this article is to analyze the narratives of family members about the suicide of older adults in Manaus, State of Amazonas, Brazil, exploring the symbolic paths that they use to make sense of this event, identifying possibilities to cope with this social and public health problem.

## METHODS

### Methodological Principles

The narrative process must be understood in its dynamic nature: past events are narrated in a way that is congruent with the current understanding of the narrator; the present is explained based on the reconstructed past^7^. Narratives should not be thought as an individual product, since the narrator uses the cultural principles and logics of the group to which he or she belongs^11^.

### Selection of Interviewees

In the *Fundação de Vigilância em Saúde* of the State of Amazonas, 37 death certificates were found for older adults (60 years or older) who committed suicide (X60-X84) in the period of 2001-2012 in Manaus. In 13 cases, we could not find the address or telephone number of family members. In order to explore in depth a smaller number of cases, we selected 12 cases of the remaining 24. The selection, for convenience, was made to ensure the characteristic diversity of the sample^14^, including older adults over 70, women, and persons who committed suicide using different methods. The family members were previously invited by letter or telephone to participate in the research^3^. At the pre-scheduled date, the family members were visited and the research objectives were detailed. The family members of eight older adults accepted to participate in the research, signing the informed consent (Tables 1 and 2).

**Table 1:** Characterization of family-narrators.

	Sex	Kinship	Age (years)	Employment
Narrative 1	Female	Daughter	44	Nursing technician
	Female	Wife	76	Housewife
Narrative 2	Female	Stepdaughter	73	Retired
Narrative 3	Male	Brother	65	Plastic artist/Street market vendor
Narrative 4	Female	Wife	60	University professor
Narrative 5	Female	Daughter	51	Seller
	Female	Ex-wife	71	Retired
Narrative 6	Female	Granddaughter	19	Student
Narrative 7	Female	Daughter	25	Student
Narrative 8	Female	Ex-wife	63	Retired
	Male	Son	45	Musician

**Table 2:** Characterization of the older adults who committed suicide, selected for the study.

Narrative	Age (years)	Sex	Place of birth	Education level	Religion	Profession	Marital status	Children	Method
1	68	M	Ceará	Incomplete elementary school	Catholic	Street market vendor	Married	6	Hanging
2	73	M	Manaus/State of Amazonas	Incomplete elementary school	Catholic	Retired	Widowed	0	Hanging
3	61	F	Manaus/State of Amazonas	Complete high school	Catholic	Unemployed	Widowed	0	Hanging
4	60	M	Manacapuru/State of Amazonas	Incomplete higher education	Catholic	Company manager	Married	4	Firearm
5	74	M	Manaus/State of Amazonas	Incomplete elementary school	Catholic	Security guard	Separated	7	Hanging
6	66	M	Juruá/State of Amazonas	Incomplete elementary school	Seventh-day Adventist	Retired	Married	6	Hanging
7	60	M	Borba/State of Amazonas	Incomplete higher education	Catholic	Research assistant	Married	3	Firearm
8	63	M	Fonte Boa/State of Amazonas	Incomplete elementary school	Catholic	Unemployed	Separated	4	Hanging

M: male; F: female

### Instrument

We used the “Semi-structured interview guide for psychological and psychosocial autopsies”, developed and validated for the investigation of suicide of older adults in Brazil^3^ to access the narrative fragments of family members about the suicide, as proposed by Owens and Lambert^18^. This guide is divided into two parts: in the first one, there are questions about the personal and socioeconomic profile and the living conditions of the older adult; in the second one, the circumstances of the suicide, precipitating factors, and the impact of the suicide on the family are discussed.

### Analytical Procedures

The interviews were audiotaped and transcribed. For each case, a metanarrative dossier was prepared, in which we compiled the narrative fragments of the family members. These dossiers were the basic texts analyzed and, from them, we elaborated the narrative synopses (Box).

**Box:** Narrative summaries.

1st narrative: He left his hometown as a young man. He worked hard as a street market vendor. He was sad, in a sense, with his children, as none wanted to pursue his business. A year before his death, he organized a trip to visit his mother in another state. He wanted to show to his mother the family he had built. Just before the trip, his mother died. Months later, after performing exams, he was informed by the doctor that he had a serious and incurable illness (pulmonary emphysema). After that, he started to have visual and auditory hallucinations, and he attempted suicide by drowning. The family tried to get him to attend a mental health service, but he declined. He committed suicide on a Sunday morning.
2nd narrative: During his old age, his wife died. Over time, he progressively lost his vision because of diabetes. Saddened, he isolated himself. He had difficulties in the relationship with his stepdaughter because he did not accept her “rebellious” behavior. The family did not think about the possibility of taking him to be evaluated in a specialized service. He was found hanged by his stepdaughter at approximately 5:30 am.
3rd narrative: When still young, she lost her husband and brother in a car accident. After years working in an industry, she was fired before retiring. After that, she was unable to return to the job market, which made her desperate. In the last days of her life, she became more sad and isolated in her room. The family did not see any need to take her to a mental health service. The older woman was considered a very fierce person, and her death was considered an act of madness. She was found hanged in her room at approximately 7:00 am.
4th narrative: He worked for many years as a manager of a company. When close to retiring, he discovered that the company was not paying his labor rights. After a lawsuit, he accepted an agreement that included demotion and lowering of the salary. He had financial and adaptation difficulties. He appeared sad and sometimes he would say, “Only if I take my life and pass it to you to finish paying.” The family did not think about the possibility of taking him to a mental health service. Suicide occurred on New Year's Eve.
5th narrative: He had not lived with his wife and children for years. The separation was motivated by his recurring involvement with other women. Such behavior alienated the older adult in relation to his relatives. Years later, his wife and children were summoned to attend a health service because the older adult had been diagnosed with prostate cancer and was in “deep depression”, having been left, without money, by his then partner. He returned to his ex-wife's house. He constantly referred to the fear of sexual impotence. The family took him to the psychiatric emergency, being indicated ambulatory follow-up that was not carried out because of his refusal. He hung himself at dawn in the bathroom.
6th narrative: During his old age he was kidnapped. He became more sad, isolated, and unable to perform his daily activities. He told his family that he could not live with fear. He was taken to an initial mental health care, but he did not accept treatment. The relatives raised the possibility that the older adult had suffered some type of sexual violence during the attack. He was found hanging from a tree at 4:30 am.
7th narrative: The older adult have always used alcohol and marijuana. This consumption intensified after leaving work because of pain associated with osteoarthritis. His work involved long trips through the interior of the state. He had a conflictive relationship with his children, who resented his recurrent and public use of the substances. Two days before his death, after drug use, he attacked his wife. The family members proposed going to a mental health service, but he did not accept it. On the day of his death, he left home saying that he would drink with his friends and say goodbye to them. The family members opted, on this day, to leave the house to avoid conflicts. In the empty house, the old man shot himself, bleeding for hours until his death. For his family members, the suicide was a form of revenge against his wife, who would not have forgiven him for the recent aggression she had suffered.
8th narrative: The older adult had not lived with his wife and children for years. The separation was motivated by his recurring involvement with other women. His work involved traveling through the interior of the state. He lost his job after suffering his first cerebrovascular accident (CVA). He returned to his old home to receive care from his former wife when he suffered a second CVA. He constantly referred to the fear of impotence. He would say, “I am here like rain,” referring to the fact that he could leave at any moment. He participated in a psychotherapy group. Before the suicide, he presented visual and auditory hallucinations. The older adult hanged himself with an electric wire tied to a window grate.

The starting point for the analysis of the material was a set of questions adapted from those proposed by Gomes and Mendonça^9^: a) who were the characters, b) how did they interact with each other, and c) how do the events narrated intertwine with each other and with the death of the older adult? In the analytic-interpretative process, we mainly tried to perform the hermeneutic double exercise^8^: to interpret the interpretation of narrators. To this end, we take into account the reports of interviewees, the interpretations of researchers, and the literature data.

Starting from the theoretical premise that suicide is a universal, complex, and multi-mediated human phenomenon^5^ ^,^ ^12^ ^,^ ^16^, we highlighted, as central references for the analysis of the material, texts that have investigated the suicide of older adults from a gender perspective^13^ ^,^ ^15^ ^,^ ^17^ ^,^ ^20^ and its correlations with the sociofamiliar context^6^ ^,^ ^19^ and with mental disorders^4^ ^,^ ^10^.

### Ethical Aspects

Research approved by the Research Ethics Committee (CAAE 30929114.8.0000.5020).

## RESULTS

### Losses

In different narratives, the starting point was the description of one or more losses suffered by the older adult (Table 3). Health-related losses were present in six of the eight narratives.

**Table 3:** Recurring themes in the narratives.

Narrative	Losses	Family conflicts	Indications of psychopathology	Regular follow-up in mental health service
1	Yes Health: pulmonary emphysema Family member: mother	Yes Children	Yes Psychosis Previous try	No Family would have tried, and the older adult refused
2	Yes Health: diabetes, blindness Family member: wife	Yes Stepdaughter	Yes Depression: isolation	No Family would not have identified the need
3	Yes Labor Family member: husband and brother	Not reported	Yes Depression: isolation	No Family would not have identified the need
4	Yes Labor	Not reported	Yes Depression: sadness and ideas of death	No Family would not have identified the need
5	Yes Health: prostate cancer, impotence	Yes Wife/children	Yes Major depression	No He would have started but abandoned
6	Yes Health: inability to perform daily activities	Not reported	Yes Depression Ideas of death	No He would have started but abandoned
7	Yes Health: osteoarthritis and pain Labor	Yes Wife/children	Yes Substance dependence/ intoxication	No Family would have tried, and the older adult refused
8	Yes Health: Cerebrovascular accident, impotence Labor	Yes Wife/children	Yes Depression Ideas of death Psychosis	No He would have started but abandoned

As in the literature^12^ ^,^ ^16^, we observed the association between suicide and chronic diseases in five narratives (1st, 2nd, 5th, 7th, and 8th). An evident aspect was the catastrophic effect that the diagnosis of these conditions can have on the older adults.

In the first narrative, shortly after the diagnosis of pulmonary emphysema and the explanation of its incurability, the older adult would have begun to present great psychopathological changes. In the fifth and eighth narratives, the older adults would have been very shaken by the fact, or the possibility, of developing sexual impotence. The way in which the diagnosis of a chronic condition affects the imaginary of older adults is perceived as relevant by the narrators, regarding their relation to suicide. In the sixth narrative, the loss of health was related to the inability to perform the activities of daily living because of a psychopathological situation that would have started after a robbery.

Losses related to work were present in half of the narratives. The literature points to the importance of job loss in the suicide of older men, giving them a sense of lack of social place and difficulties to honor debts and manage time^17^. In the seventh and eighth narratives, the older adults, because of illness, were removed from their work, typical of the Amazonian context, characterized by its long and constant trips to municipalities within the state. These activities gave them a peculiar way of life, with greater freedom and less possibility of family censorship for the behaviors considered as deviant (alcohol and drug consumption, seventh narrative; sexuality, eighth). The absence of these activities also meant the loss of this lifestyle.

In the fourth narrative, the older adult would have discovered that he could not retire because of the lack of payment of social security by the company after a long time working there, in a role of relative prominence. He would have agreed to work in a much more modest position while solving the pendency, a situation that greatly distressed him. Although this older adult continued to work, there was a significant loss of salary and status, associated with feelings of humiliation and helplessness, which were identified as important factors in understanding the relationship between suicide and work in older adults^17^.

In the third narrative, we have the description of the suicide of an older woman who would have been fired when old. Without being able to return to the labor market, she began to present economic difficulties. In the case of suicide of older women, the literature usually emphasizes the difficulties associated with the exercise of the functions traditionally related to the feminine condition, such as the roles of wife and mother^15^. In the case of this narrative, we need to look at the complexity of the interrelationship of the different losses experienced by the older woman. In her youth, she would have lost her husband (and a brother), and she did not remarry or had children. By choice, or because of destiny, she ended up not adhering to the female hegemonic condition, of wife-caregiver. Work, in this context, would have a centrality in the life of this older woman, and her loss acquired great dimensions, and could thus be associated with her option to take her life^20^.

In the first and second narratives, we have reports of family losses that occurred in old age, close or simultaneous to the occurrence of health-related losses. In the first narrative, there is the report of the death of the mother of an older migrant, who, as a young man, left his native land and with many difficulties would have achieved relative professional and family success. There is evidence of the association between suicide and migration^1^. Old age is a moment in which “the memories of the past are revived, and those who have distanced themselves in time and from the native land miss them”^19^ (p.1706). The death of the mother occurred on the eve of a trip in which he intended to take his family to his land and introduce them to his mother. This trip would be a type of accountability for him, his children, and his mother. However, it did not happen, causing deep sorrow to the older adult. Following this event, he would have received the diagnosis of a serious and incurable condition (already discussed). Sometime later, the older adult tried to take his life. In the second narrative, there is the report of the loss of the wife, of a lifetime, of a childless older adult who was going blind, and who would have conflicts with a stepdaughter. There is a great amount of epidemiological data linking widowhood to suicide^16^. What these studies do not reveal is the context in which this widowhood would occur, which can occur amidst helplessness, illness, and family conflicts, further making the older adults vulnerable to suicide.

### Family Conflicts

Recurring narratives describing conflicts involving children or stepchildren were attempts by the older adult to discipline or influence their behavior. The first narrative addresses the frustration of the older adult because none of his children wanted to work in the humble and arduous business that has guaranteed the support of the family for years. In the second narrative, the older adult would not accept the sexual orientation and use of psychoactive substances by the stepdaughter.

In the fifth and eight narratives, we can observe older adults with a history of conflicts with their wives (or ex-wives) and children because of their involvement with other women. Both narratives relate the drama of the older adults who have left their homes because of these conflicts, but who had returned after losing their ability to self-care. This situation, marked by contradictions, is described as related to suicidal events among older adults^13^.

In the seventh narrative, we have a situation of an older adult whose family members resented his heavy and public use of psychoactive substances. The discomfort of the family in relation to this practice was intensified after the older adult had stopped working, when the use came to be no longer exclusively in the context of business travel.

It is implicit in the narratives the idea that the authoritarian thinking of older adults would be at the genesis of family conflicts. It is also implied the frustration of older adults with the weakening of their masculine authority because of old age, illness, and other losses. The family conflict and the suicide of the older adult would be related to a type of malaise derived from the incapacity of the older adult to impose his weakened authority over the will of others^17^. In these narratives, the older adult would be responsible for family tensions.

A recent study with older adults who survived suicide attempts shows a more explicit relationship between suicide and family conflicts when compared to studies that used psychosocial autopsies, in which relatives of older adults who committed suicide were interviewed^19^. This study highlights the importance of the feelings of abandonment, incomprehension, resentment, as well as the absence of expressions of affection, respect, support, and even explicit violence by family members in the speeches of older adults who survived the suicide attempt.

The difference between psychosocial autopsy studies and the studies that interviewed the survivors is not only related to the fact that the first ones are cases of consummate suicide and the second ones case of attempts. They are also distinguished by the fact that they are studies that have different points of view. In the first case, the family members are the focus; in the second, the survivors. Such epistemic vigilance is necessary, since the report of the interviewee cannot simply be considered as a fact. It will always be a version of the facts, committed to the social and symbolic place of the one who issues and omits points of view. We emphasize the need to consider the possibility of much more complex relationships between the family conflicts and the suicide of older adults than those presented by the family members. Special contextualization should be done in relation to the point of view that makes the older adults responsible for the conflicts. This would not mean disregarding that personal idiosyncrasies, difficulties or not of the aging in itself, can contribute to the genesis and maintenance of family conflicts that would be related to the suicide of older adults.

### Indications of Psychopathology and Access to Health Services

In the narratives, after the older adult experienced losses and sometimes experienced a family conflict, behavioral changes appeared that preceded the suicidal act, which we call indications of psychopathology. The narrators sometimes reported that they did not immediately identify these changes, only noticing them during the hard process of thinking and reviewing what had occurred.

It is necessary to point out that we cannot make inferences about specific psychiatric diagnoses considering the strategies used here. On the other hand, we can consider associations between possible psychopathological situations and the search for care in the field of mental health, which is relevant since, in the narratives, none of the older adults would be in treatment or follow-up in psychosocial care services.

Evidence of depressive symptoms (sadness, discouragement, isolation, ideas of death) could be observed in five narratives (2nd, 3rd, 4th, 5th, 6th). In three of them (2nd, 3rd, and 4th), the family would not have identified the need to take the patient to treatment, and in the other two (5th and 6th), the older adults would have started treatment but abandoned them because they did not think it was necessary.

In turn, psychotic symptoms (hallucinations or delusions) were identified in two narratives (1st and 8th). In both cases, the family would have tried to take the patient to specialized care: in the first narrative, the older adult would have refused, and, in the second one, the older adult would have started but abandoned the treatment. In the seventh narrative, we can observe indications of problems related to the use of alcohol and other substances.

It was exclusively in the narratives of the older adults who presented evidence of depressive situations that occurred reports of families that would not have noticed the need to seek care. Research studies point to the relationship between depression and suicide in older adults^5^ and the effectiveness of adequate treatment of these situations in the prevention of these deaths^4^. Factors such as atypical clinical presentation and the belief that the sadness of the older adult in the face of losses that would accompany the aging process would invariably be normal, make it difficult to diagnose depressive disorders, including by specialists^10^. We can observed that the narrators are not experts, but the idea of being unable to perceive the behavioral changes before the suicide is something that torments the thoughts of the family members^6^. Thus, we must emphasize the importance of trying not only to identify (and treat, if necessary) depressive disorders in older adults but also relativize the inability and self-blame of family members in not realizing possible depressive changes in the older adults.

Psychotic symptoms, given their own eccentricity, are usually noticed. When it occurs in older adults, without previous psychiatric symptoms, apparently as in the case of the first and eighth narratives, there is a need to exclude the possibility of psychosis being caused by general medical illnesses, such as those described in the cases in question.

The characteristic of psychotic situations is the inability of the subject to perceive that their behavior and perceptions are altered. In the narrative cases, treatment would not have been kept or even started because of the refusal of the older adult. This treatment in a refusal context is challenging, both logistically and morally, not only for family members, but also for health services.

The use of substances (and the problems associated with them) is a complex and prevalent situation. Sometimes represented as a character problem, there is, in different situations, resistance in resorting to health services. The occurrence of explicit violence, as described in the seventh narrative, can be a turning point for family members to use these services. Initially, substance use appears as a characteristic of the older adult, carried out in the context of work trips and interfering little in the routine of the family. With illness and lack of work, consumption intensifies, becomes closer to the family, and is associated with violence. The theme of violence is then impregnated in the narrative. Violence motivated the search for treatment for the older adult. The older adult killed himself violently, bleeding all night. The suicide would also have been an act of violence against the wife, a revenge, for not having forgiven him, after the previous aggression. This narrative helps us to get closer to the complex relationships between the suicide of older adults and substance use, and the losses related to health, work, and violence. It also helps to think how shocking is the violence within families, motivating actions (search for treatment) and setting up representations about death and dying.

## FINAL CONSIDERATIONS

From the analysis of the narratives, we can propose that family members would conceive the suicide of the older adults as related to losses, which would occur in a strained sociofamiliar scenario, leading to the appearance of psychopathological situations that, if not properly followed, would result in death. There would also be something inexorable in this sequence of events. The older adults, by the very time of their life, would tend to accumulate losses of different aspects in their trajectory. Their rigor and other relational limitations would both favor family conflicts and hinder adherence to treatment.

This symbolic model, which has wide support in the hegemonic medical-psychological discourse, in a sense minimizes possible self- or hetero-acusations directed at family members. It is widely recognized in the literature the feelings of guilt that follows family members, as well as the stigmatization that they sometimes experience^16^ ^,^ ^19^. This model seems so strong and coherent that, when it is not widely present in particular cases, it seems to require complementary explanatory strategies.

In the third narrative, we have the older woman who had lost her husband (and brother) in her youth and her job in her old age, and she progressively became isolated without the family realizing it. In the sixth narrative, we have an older person with great functional losses related to the psychopathological condition that was developed after a robbery and for which he did not accept specialized treatment. In both situations, there were no reports of family conflicts. In the case of the third narrative, suicide is seen as a bizarre, incomprehensible, and explicit case of madness. We can observe that, although in all cases there is mention of psychopathological changes, sometimes subtle, in no other situation there was an explicit reference to madness. In the sixth narrative, it is used the possibility of the older adult being a victim of sexual violence during a robbery, which is something that would deeply and irreversibly shake the honor of the older adult. However, this is very unlikely to have occurred in our interpretation. Thus, in the incompleteness of the explanatory system, something imponderable would have to be added to make sense of the suicide.

In addition, the analysis of the narratives also indirectly provides elements to elaborate some recommendations for the confrontation of suicide in older adults, considering the point of view of the family members. Special attention should be given to identify the older adults who present losses, family conflicts, and signs of psychopathology and who do not follow-up psychosocial care services. It would be important the qualification of health professionals in how they communicate to older adults the occurrence of chronic and incurable conditions, recognizing the importance of how socio-cultural and psychological factors will affect the way this news will be apprehended.

In addition, strategies to help older adults cope with family conflicts and losses, associated or not with the aging process, as a way of empowering them, should be developed and made available by intersectoral actions. The adequate treatment of psychopathological conditions should be implanted in a context in which active search mechanisms also existed for older adults who have abandoned follow-up.

The implementation of these actions is a large but necessary challenge to be faced in Manaus, where there is a low availability of psychosocial care services, which are not articulated with specialized care services in tertiary medical conditions, with low coverage (less than 40%) by the basic health care.

Finally, one last aspect to be considered is the fact that the interviewees were mostly women, who analyzed the circumstances of the death of older men. This fact certainly influenced the findings of this research. Thus, we recommend new studies, with other sample compositions, as we understand that they will certainly help deepen the knowledge about the ways in which family members give meaning to the suicide of older adults.

## Footnotes

a Instituto Brasileiro de Geografìa e Estatística. Sistema IBGE de Recuperação Automática - SIDRA: banco de tabelas estatísticas. Rio de Janeiro; 2015 [cited 2016 Mar 20]. Available from: http://www.sidra.ibge.gov.br

b Costa ALS. Suicídio de idosos no município de Manaus, Amazonas [dissertation]. Manaus: Programa de Pós-graduação em Saúde, Sociedade e Endemias na Amazônia da Universidade Federal do Amazonas; 2015.
